# Nutrition and Physical Activity Behavior in 11–14-Year-Old Schoolchildren in Serbia

**DOI:** 10.3390/children8080625

**Published:** 2021-07-23

**Authors:** Biljana Cvetković, Milan Cvetković, Tanja Petrušič, Višnja Đorđić, Saša Bubanj, Boris Popović, Slobodan Andrašić, Svetlana Buišić, Špela Bogataj

**Affiliations:** 1 Institute of Food Technology in Novi Sad, University of Novi Sad, Bulevar Cara Lazara 1, 21101 Novi Sad, Serbia; biljana.cvetkovic@fins.uns.ac.rs; 2 Faculty of Sport and Physical Education, University of Novi Sad, 21101 Novi Sad, Serbia; cveksha@gmail.com (M.C.); djordjicvisnja@gmail.com (V.Đ.); borispopovic0803@gmail.com (B.P.); 3 Faculty of Education, University of Ljubljana, 1000 Ljubljana, Slovenia; tanja.petrusic@pef.uni-lj.si; 4 Faculty of Sport and Physical Education, University of Niš, 18000 Niš, Serbia; bubanjsasa@yahoo.co.uk; 5 Faculty of Economics, University of Novi Sad, 24000 Subotica, Serbia; slobodan.andrasic@ef.uns.ac.rs; 6 Faculty of Education in Sombor, University of Novi Sad, 21101 Novi Sad, Serbia; svetlana.buisic@pef.uns.ac.rs; 7 Department of Nephrology, University Medical Centre, 1000 Ljubljana, Slovenia; 8 Faculty of Sport, University of Ljubljana, 1000 Ljubljana, Slovenia

**Keywords:** nutrition, physical activity, student, primary school, gender, age differences

## Abstract

Regular physical activity and healthy diet have a significant positive impact on children’s health. Lack of physical activity increases the risk of various diseases, while obesity has become an alarming health problem worldwide. The aim of this study is to investigate the patterns of physical activity and diet among 11–14-year-old school children in Serbia. The sample included 623 primary school children, of whom 333 were boys (53.45%) and 290 were girls (46.55%). The children were also divided according to their age/grade: fifth grade/11 years (24.40% (*n* = 152; M = 84, F = 68)), sixth grade/12 years (25.68% (*n* = 160; M = 91, F = 69)), seventh grade/13 years (26.81% (*n* = 167; M = 83, F = 84)) and eighth grade/14 years (23.11% (*n* = 144; M = 75, F = 69)). Children’s lifestyle was assessed using two subscales of questionnaires based on the Health-Promoting Lifestyle Profile model II. The Mann–Whitney U test showed no statistically significant difference between boys and girls in the variables Nutrition (*p* = 0.81) and Physical Activity (*p* = 0.91). The Kruskal–Wallis test was applied and showed no statistically significant differences between children of different ages, regardless of gender, in the variable Nutrition (*p* = 0.63). However, differences were evident in the variable Physical Activity (*p* < 0.001), with the highest mean scores recorded in 12- and 13-year-old children and the lowest in 14-year-old children. Additionally, no statistically significant differences were found among boys as a function of age in the variables Nutrition (*p* = 0.55) and Physical Activity (*p* = 0.15), nor among girls in the variable Nutrition (*p* = 0.43), but in the variable Physical Activity (*p* = 0.01). In general, it can be concluded that the older primary school children have relatively satisfactory dietary and physical activity habits, but that these behaviors decrease with age, especially the level of physical activity. There is a need for better and more effective education of children about the benefits and importance of proper nutrition and regular physical activity.

## 1. Introduction

In 2019, 18% of children aged 5–19 and 6% of children under five were overweight or obese globally [1]. In addition, 340 million children and adolescents aged 5–19 years were overweight or obese [2]. Recent international and national reports have pointed to the growing epidemic of obesity among school-age children, particularly in the last three decades among children aged 6–19 years [3].

Smartphones and tablets, including software applications, have become an integral part of children and adolescents’ lives. In developed countries worldwide, approximately 70–80% of adolescents regularly use a smartphone [4]. Such a lifestyle may result in reduced physical activity levels and is associated with several obesity risk factors among children and adolescents [5,6]. Active commuting to school has decreased in some countries [7,8] further contributing to a sedentary lifestyle. Childhood health behaviors can influence adolescent health behaviors, with sedentary behaviors being of a particular relevance [9]. This inactive lifestyle and increased consumption of unhealthy, fast food have contributed to the development of several associated diseases, the most common being non-communicable chronic diseases [10,11,12]. According to a multi-center, multi-country study, a small percentage of the adolescent population (approximately 10%) is involved in activities that can ensure good physical condition in addition to exclusive health aspects [13]. Boys tend to be more active than girls, and physical activity declines with age [14,15,16,17].

During adolescence, there is an increased need for energy due to physical changes and intense physical and mental activity [18]. Regular physical activity and healthy diet are related to numerous health outcomes in children and adolescents, including improved physical fitness and cardiometabolic health, weight control, prosocial behavior, mental health, cognitive outcomes etc. [19,20,21]. Several studies show that obesity is associated with increased time spent in sedentary activities and high intake of energy-dense, nutrient-poor foods such as soft drinks, salty chips, sweet cookies, and candy [22,23]. On the other hand, regular physical activity and a diet rich in fruit, vegetables, legumes, and whole grains have been shown to be protective [24,25].

Regarding dietary habits, recent research based on a representative sample of children in Serbia showed that children and adults do not consume enough fruit and vegetables in their daily diet; they use excessive amounts of fatty foods and their diet contains too many foods with high sugar content; their regular diet does not contain enough wheat, milk, and dairy products and they rarely read the labels on food products when buying them. In addition, a small proportion of the population has regular physical activities, be it sports, recreational activities or transportation to school or work [26].

An active physical life contributes to optimal development and improvement of physical, mental, emotional and social health [27]. Another significant factor for a healthy life is nutrition, which has unfortunately deteriorated significantly in recent decades and young generations acquire bad habits early in their lives [28]. Due to the positive impact on health, it is important to increase the activity levels and participation of young people who are least active [29] and this should be a goal of community and school-based health promotion interventions.

Monitoring the nutritional status and health habits of adolescents and children has many benefits for schools and parents as it indicates the adequacy of growth and development and later helps to understand the current status; there are many things that can be done to improve the quality of children’s nutrition and physical activities during school years [30,31]. This requires comprehensive and multi-sectoral interventions to improve healthy eating and increase levels of physical activity among primary school-aged children [32].

Inadequate physical activity, poor dietary habits, and sedentary lifestyle can cause various changes in children’s growth and development. Therefore, this research provides insight into the lifestyle of children and adolescents in Serbia, in terms of health-related behaviors related to nutrition and physical activity.

## 2. Materials and Methods

The study was conducted as a cross-sectional study. Data were collected using the two subscales of the Health-Promoting Lifestyle Profile (HPLP-II) questionnaire [33]. A multistage sampling procedure was applied, combining cluster and simple random sampling. The final sample included 623 middle school-age children (333 boys (53.45%) and 290 girls (46.55%)), aged 11 to 14 years. The participants resided in rural areas or small towns from Serbia-North and Serbia-South NUTS 1 regions.

Dependent variables in the study were the scores obtained on the subscales:

(1) Nutrition (mean score) and
(2) Physical Activity (mean score).

Gender and age (school level) were considered to be independent research variables.

The children’s lifestyle was assessed by two subscales of HPLP questionnaire [33]. The whole questionnaire contains 52 items divided into 6 subscales, the first two with a total of 17 items were used for the study. One subscale related to diet (9 questions) and the other related to physical activity level (8 questions).

The statements from the subscales relate to different habits regarding physical activity and diet. An example of an item from the diet subscale is, “I chose a low-fat diet.” An example of an item from the Physical Activity subscale is: “I participate in a planned exercise program (e.g., sports or dance).” For each statement offered, the respondent could choose one of four responses offered: Never—1, Sometimes—2, Often—3, Routinely—4. The subscale score is calculated as the respondent’s average score obtained after dividing the total score by the number of questions in each subscale.

The survey was conducted in the classrooms in 2019 and 2020 after gaining approval from the principals. Parents gave written consent for their children to participate in the survey in accordance with the requirements of the Declaration of Helsinki.

The children completed the questionnaires at the beginning of physical education classes, following the standard instructions of the investigators. There was no time limit for completing the questionnaires and the average completion time was approximately 10 min. When collecting the questionnaires, the survey officers checked if the children had completed the entire questionnaire properly.

Descriptive statistics were used to process the research data. Mean and standard deviation (SD) were calculated for the nutrition and physical activity subscales. The deviation of the results from the normal distribution at the borderline of significance was tested using the Kolmogorov–Smirnov test on the whole sample at the *p* < 0.05 statistical significance level.

The Mann–Whitney U test was used to determine the differences between the groups as some variables were found to deviate from the normal distribution. To determine the difference between the groups formed according to age, the Kruskal-Wallis test was performed at the significance level of *p* < 0.05, considering the identified deviation of the results from the normal distribution in most of the groups of the analyzed variables when classified by gender and age. All data were processed using statistical program IBM SPSS version 20.

## 3. Results

### 3.1. Descriptive Statistical Analysis of the Whole Sample

The total sample of 623 middle school children was divided into clusters by age: 11-year-old students (24.40% (number (*n*) = 152; male (M) = 84, female (F) = 68)); 12-year-old students (25.68% (*n* = 160; M = 91, F = 69)); 13-year-old students (26.81% (*n* = 167; M = 83, F = 84)), and 14-year-old students (23.11% (*n* = 144; M = 75, F = 69)).

The values of the descriptive statistics of the whole sample, before all the values of the coefficient of variation, showed moderate homogeneity of the results in the Nutrition subscale and low relative heterogeneity of the results in the Physical Activity subscale. The Physical Activity subscale reached absolute minimum and maximum scores, which is not the case for the Nutrition subscale. The average score of the Physical Activity subscale (2.80) is higher than the average score of the Nutrition subscale (2.58). Both scores are lower than the average scale score. Based on the statistical significance of the Kolmogorov–Smirnov test (KSp) in Table 1, a deviation of the results from the normal distribution can be observed for both variables (KSp < 0.001).

The item analysis in Table 2 showed that the largest percentage of children sometimes choose fat-free foods (60.51%). The largest percentage of children sometimes (39.96%) use sugar and sweets. As many as 53.13% children reported that they never eat 6 to 11 servings of bread and cereal products per day. A total of 39.81% of children routinely eat 2 to 4 servings of fruit per day1.90% of children sometimes eat 3 to 5 servings of vegetables per day. A total of 33.06% of children routinely eat 2 to 3 servings of milk, cheese, and yogurt per day. The largest percentage of children sometimes eat up to 2–3 servings of meat and eggs per day—36.12% of them. The largest percentage of children, almost half, never read labels on nutrients, fats and salts (42.0%). As many as 91.01 children routinely eat breakfast.

Regarding physical activity, 50.08% children routinely participate in organized exercises, 20.23% sometimes do so, 19.10% often do so, while 10.59% children never participate in such activities. A total of 48.80% of children routinely engage in vigorous exercise for 20 min or more at least three times a week, 26.48% do so often, 17.82% sometimes and 6.90% never.

As for participating in moderate physical activity (MVPA) 30–40 min, 5 or more times a week, 36.68% of children do so routinely, 30.50% often, 24.40.1% sometimes and 6.42% never. A total of 33.39% of children routinely participate in leisure time physical activities, 27.61% sometimes, 25.84.1% sometimes and 13.16% never. The largest percentage participants routinely do stretching exercises at least 3 times a week (42.22%), while 10.59% never do so, 24.72% sometimes and 22.47% often. A total of 72.07% of children routinely do stretching exercises during usual daily activities, 24.72% of them sometimes, while 22.47% often do so. A total of 10.59% children never get such exercise. As many as 56.34% children never check their pulse rate during exercise, 6.90% do it routinely and 11.72% do it often. About a quarter of children sometimes check their pulse (25.04%). A total of 35.32% of children never reach their target pulse rate while exercising, 10.01% do so routinely, 22.47% often, and 32.10% sometimes.

### 3.2. Analysis of Descriptive Statistics and Differences between Gender Groups

The values of the descriptive statistics, taking into account the gender of the children (Table 3), have shown the heterogeneity of the results in the variable Physical Activity in both analyzed subsamples. Considering the statistical significance of the Kolmogorov–Smirnov test (KSp), a deviation from the normal distribution of the results in both subsamples of the two analyzed variables Nutrition and Physical Activity (KSp < 0.001) was found (Table 2).

The results of the Mann–Whitney U test show that there are no statistically significant differences between boys and girls in Nutrition (*p* = 0.81) and Physical Activity (*p* = 0.91) subscales (Table 3).

### 3.3. Analysis of Descriptive Statistics and Differences between Age Groups

Students also achieved lower mean scores in the Nutrition subscale compared to the Physical Activity subscale, with moderate age variation: 2.59 for 11-year-old students, 2.62 for 12-year-old students, 2.54 for 13-year-old students, and 2.56 for 14-year-old students. Mean scores in the Physical Activity subscale range from 2.70 for 14-year-olds to 2.87 for 11- and 12-year-olds, representing a variation of 0.17 points. The mean score for 13-year-olds was 2.73 (Table 4).

The results of the Kolmogorov–Smirnov test showed a deviation from the normal distribution of scores in both variables analyzed in all four subsamples (Table 4).

Considering the deviation from normal distribution in the analyzed variables in the respondent groups, the Kruskal–Wallis test was conducted (Table 3). It can be concluded that there are statistically significant differences between the children of different ages in the Physical Activity variable (*p* < 0.001), but not in the variable Nutrition (*p* = 0.63).

The students aged 11 and 12 years are the most active, followed by the students aged 13 years, while the students aged 14 years are the least active, indicating that the level of physical activity decreases with age, in a linear slope. The older the primary school students were, the less physical activity was reported.

Reviewing the obtained results in the analyzed variables (Table 5), in the group of boys, it can be noted that the average scores on the Nutrition subscale ranged from 2.55 in the 11- and 13-year-old children to 2.64 in the 12-year-old children. The children aged 14 years had a score of 2.56. The mean scores in the Physical Activity subscale range from 2.70 in the 14-year-old subjects to 2.85 in the 12-year-old subjects, which corresponds to a zone of unhealthy habits.

The scores of the Kolmogorov–Smirnov test (Table 4) have shown deviations from the normal distribution in the variable Nutrition in the 12- and 13-year-old children (KSp < 0.001), including the variable Physical Activity in the 11-year-old children (KSp = 0.01). No significant deviations from the normal distribution were found in the other school grades (SVp > 0.05).

Considering the observed deviation from the normal distribution in the analyzed variables in most boys educated by age, the test Kruskal–Wallis was conducted (Table 4). There are no statistically significant differences between boys of different ages in the variables Nutrition (*p* = 0.55) and Physical Activity (*p* = 0.20).

Considering the obtained results in the analyzed variables Nutrition and Physical Activity in the group of girls (Table 6), we can notice that they also obtained higher average scores in the Physical Activity subscale than in the Nutrition subscale, with moderate age differences: 2.92 in 11-year-olds and 2.89 in 12-year-olds, with 2.71 in 13-year-olds and 2.69 in 14-year-olds. Mean scores on the Nutrition subscale ranged from 2.53 for 13-year-olds to 2.62 for 11-year-olds, representing a variation of 0.19 points.

The scores of Kolmogorov–Smirnov test have shown deviation from normal distribution of scores in Nutrition variable in girls (Table 6), 12, 13, and 14 years old. (KSp < 0.001). Deviations from normal distribution were found in the variable Physical Activity in two subsamples of girls, especially at the age of 11 and 12 years old (KSp < 0.001). No statistically significant deviations from the normal distribution were found (KSp > 0.05).

To determine the differences in the subscales, the Kruskal–Wallis test was performed (Table 6). It can be noted that there are some statistically significant differences between girls of different ages in the Physical Activity variable (*p* = 0.01), but this is not the case in the Nutrition variable (*p* = 0.43).

## 4. Discussion

The study was conducted on the population of middle school students in Serbia, with the aim of determining the patterns of diet and physical activity. Average sample scores (*n* = 623), indicate relatively satisfactory dietary and especially physical activity patterns, considering that the average scores were moderately higher than the average scale score (2.50).

The sample was fairly consistent, but a significant effect of age and a combined effect of age and gender on dietary and physical activity patterns was found. This means that middle school children belong to different target groups depending on age, dietary, and physical activity habits. This is not the case for groups formed by gender. In terms of gender, no statistically significant differences were found in levels of physical activity and dietary habits, finding of previous studies employing similar samples of subjects [34,35,36], who confirmed that there are no differences between adolescent children in terms of macronutrient intake and food consumption, i.e., that they have similar dietary habits. In contrast to the results of the study conducted among the children and adolescents from Europe (HELENA study) [37], the current study showed that the children consume significantly less nutrients and have a much worse lifestyle when it comes to physical activity and eating habits.

The highest percentage of children routinely engage in sports or participate in moderate physical activity, which is a good indicator of healthy lifestyle among middle school children. Although different questionnaires were used, such conclusions can be drawn considering that children in HELENA study were reported to be active in moderate physical activity on average 540–600 min/week, while less than 1/3 of children from Serbia participate in regular physical activities of the same type.

Compared to the results of Ostojić et al. (2017) [26], conducted on the nationally representative sample, middle school students in Serbia in the present study achieved similar mean scores in the Nutrition variable (2.58 compared to 2.59) and higher mean scores in the Physical Activity variable (2.80 compared to 2.68). The students who participated in this research showed a more favorable behavioral pattern in diet and a better one in physical activity. Such results are comparable with the research results of Đorđić et al. (2020) [38], where a different methodology was used, but it also showed that children in Serbia have relatively good health habits in terms of nutrition and physical activity. Since we did not consider family or SES background in details, it is hard to determine if children’s health behaviors are driven solely by their own motivation.

Item-by-item analysis showed that the daily diet of the adolescent population, regardless of gender and age, does not include enough cereals, milk, and dairy products. The participants reported almost not reading nutritional labels at all. Although most of the food is purchased by parents/caregivers, children in Serbia usually choose and buy some snacks by themselves, or go to shopping with adults, so education on food labeling might be an effective health-promoting strategy. Even though the scores on Nutrition and Physical Activity subscales are average, we can still confirm some quite good health habits of children when it comes to the intake of nutrients, especially fruit and vegetables, meat, and regular breakfast. The participants reside in rural and semi-urban areas which might explain availability of fresh fruit, vegetables, and meat.

In addition, a relatively high percentage of the surveyed population routinely participates in physical activity, whether in the form of sports, recreational activities, sufficient walking or cycling, which may be related to living in less urban environment. In early adolescence, the manifestation of physical activity level is strongly influenced by the environment and children’s participation in sports, which was also confirmed by the findings of Chen et al. (2018) [39], who claimed that children who were more active in school and extracurricular activities had significantly higher levels of moderate physical activity on a weekly basis.

The significantly lower level of physical activity among middle school students, regardless of gender, may be a result of lost interest in physical activity during adolescence, when they are usually engaged in other subjects and interests, with many children leaving sports at this age. A very small percentage of children participate in sports on a regular basis. A small percentage of children routinely stretch, three times a week, and even if they are active in some way, this level is currently insufficient to meet the health habits required for proper growth and development. Since in Serbia physical education is an obligatory school subject, provided three times per week for all children in middle school, the results might reflect ineffective physical education delivery.

The research findings did not show statistically significant differences between adolescents of different genders, but that there are age differences when it comes to diet and exercise behaviors. Eleven- and twelve-year-old students had higher mean scores on the variables studied compared to 13- and 14-year-olds, especially in girls, which may indicate a trend that health habits worsen with age, more so in girls who enter puberty earlier in comparison to boys). These findings confirm those of other researchers who reported a decline in physical activity with age, with the greatest decline occurring during adolescence [40]. The relative heterogeneity of the results at all ages in the two variables studied, and especially in the Physical Activity variable, is a consequence of the fact that the sample includes the students who actively participate in daily sports and training, who adhere to the plan and diet during a certain period of time; however, there were also children who do not participate in organized sports, who are not physically active and who exercise much less. They also skip meals, eat unhealthy diets, avoid grains and dairy products while consuming large amounts of carbohydrates. All of this affects the average values of the above variables, because the level of physical activity and sport decreases with age, especially in the upper grades of middle school. It should also be taken into account the fact that the choice of leisure or sports activities is influenced by various factors, including personal interests, family influence, and environment. This was also confirmed by the results of Chen and Zhu’s (2005) [41] research, which examined the influence of personal, school, and family variables on children’s interests. The results showed that personal interests have an influence on children’s activities, but the influence of family members, environment, i.e., school, is predominant, along with physical education during the week, teacher experience, and neighborhood safety. The analysis showed that given the dominant influence of the family and the school environment, it is necessary to invest in the quality of physical education in school.

Our results provide an insight into the current physical activity and dietary patterns of middle school students in Serbia. Based on the study results, it is possible to develop specific recommendations regarding students’ nutrition and physical activity, as well as profiling effective educational programs. Physical and health education teaching, which requires well-trained and motivated teachers, should play the key role in lifelong health education. A significant step towards creating a link between the university and everyday school practice was achieved by presenting the results of the research to the teachers and professional services involved, i.e., the schools whose students participated in the research. Promoting healthy lifestyles through regular physical activity and proper nutrition in childhood and adolescence while taking professional preventive measures to reduce overweight/obesity is critical to public health. Regular physical activity should be part of a daily life, combined with an appropriate diet. Middle schools are probably the most appropriate places to promote adequate nutrition and regular physical activity, and they can also promote parental and broader community engagement. Regular physical activity by adolescents, along with improving their nutrition, is a worthwhile investment in the health of future generations.

## Figures and Tables

**Table 1:** Analysis of descriptive statistics conducted on the entire sample.

Variable	*n*	min	max	mean	SD	KSp
Nutrition (points)	623	1.22	3.78	2.58	0.41	<0.001
Physical activity (points)	623	1.00	4.00	2.80	0.55	<0.001

Abbreviations: *n*, number of subjects; SD, standard deviation; KSp, Kolmogorov–Smirnov test.

**Table 2:** Distribution of children’ answers to HPLP questionnaire.

Variables	Never	Sometimes	Often	Routinely
1. Chose a diet that is low in fat	13.48%	60.51%	21.83%	4.18%
3. Limit use of sugars and food containing sugar	11.56%	39.96%	29.70%	18.78%
5. Eat 6–11 servings of bread and cereal each day	53.13%	30.82%	10.43%	5.62%
7. Eat 2–4 servings of fruit each day	4.49%	25.84%	29.86%	39.81%
9. Eat 3–5 servings of vegetables each day	14.92%	41.90%	24.08%	19.10%
11. Eat 2–3 servings of milk, cheese, and yogurt each day	9.31%	30.18%	27.45%	33.06%
13. Eat up to 2–3 servings of the meat and eggs each day	13.00%	36.12%	30.18%	20.70%
15. Read labels to identify nutrients, fats, and sodium content	42.70%	31.94%	12.84%	12.52%
17. Eat breakfast	0.32%	3.85%	4.82%	91.01%
2. Follow a planned exercise program	10.59%	20.23%	19.10%	50.08%
4. Exercise vigorously for 20 or more minutes at least three times a week	6.90%	17.82%	26.48%	48.80%
6. Take part in MVPA * 30–40 min, 5 or more times a week	6.42%	24.40%	30.50%	36.68%
8. Take part in recreational physical activities	13.16%	27.61%	25.84%	33.39%
10. Do stretching exercises at least 3 times per week	10.59%	24.72%	22.47%	42.22%
12. Get exercise during usual daily activities	2.72%	8.67%	16.53%	72.07%
14. Check my pulse rate when exercising	56.34%	25.04%	11.72%	6.90%
16. Reach my target heart rate when exercising	35.32%	32.10%	22.47%	10.01%

Abbreviations: MVPA *, Moderate to vigorous physical activity.

**Table 3:** Analysis of descriptive statistics and differences between gender groups.

Variable	M (*n* = 333)		F (*n* = 290)			
	Mean ± SD	KSp	Mean ± SD	KSp	U	*p*
Nutrition (points)	2.52 ± 0.42	0.00	2.57 ± 0.39	<0.001	47739.50	0.81
Physical activity (points)	2.79 ± 0.55	0.00	2.80 ± 0.54	<0.001	48036.00	0.91

Abbreviations: *n*, number of subjects; M, male; F, female; SD, standard deviation; KSp, level of statistical significance in Kolmogorov–Smirnov test; U, value in Mann–Whitney U test; *p*—level of statistical significance in Mann–Whitney U test.

**Table 4:** Analysis of descriptive statistics and differences between age groups.

Variable	11 years (*n* = 152)		12 years (*n* = 160)		13 years (*n* = 167)		14 years (*n* = 144)				
	Mean ± SD	KSp	Mean ± SD	KSp	AM ± SD	KSp	Mean ± SD	KSp	χ^2^	*p*	df
Nutrition (points)	2.59 ± 0.44	0.03	2.62 ± 0.40	<0.001	2.54 ± 0.40	<0.001	2.56 ± 0.38	<0.001	1.73	0.63	3
Physical activity (points)	2.87 ± 0.57	<0.001	2.87 ± 0.52	<0.001	2.73 ± 0.52	0.02	2.70 ± 0.54	0.03	0.63	<0.001	3

Abbreviations: *n*, number of subjects; M, male; F, female; SD, standard deviation; KV, coefficient of variation; KSp, level of statistical significance of Kolmogorov–Smirnov test; χ^2^, value chi squared test (Kruskal–Wallis test); *p*, level of statistical significance Kruskal–Wallis test; df, degree of freedom.

**Table 5:** Analysis of descriptive statistics and analysis of age-related variations among boys.

Variable	11 years (*n* = 84)		12 years (*n* = 91)		13 years (*n* = 83)		14 years (*n* = 75)				
	Mean ± SD	KSp	Mean ± SD	KSp	Mean ± SD	KSp	Mean ± SD	KSp	χ^2^	*p*	df
Nutrition (points)	2.55 ± 0.47	0.20	2.64 ± 0.42	<0.001	2.55 ± 0.40	<0.001	2.56 ± 0.38	0.06	2.09	0.55	3
Physical activity (points)	2.84 ± 0.58	0.01	2.85 ± 0.57	0.07	2.76 ± 0.50	0.20	2.70 ± 0.55	0.20	5.27	0.15	3

Abbreviations: *n*, number of subjects; M, male; F, female; SD, standard deviation; KV, coefficient of variation; KSp, level of statistical significance of Kolmogorov–Smirnov test; χ^2^, value chi squared test (Kruskal–Wallis test); *p*, level of statistical significance Kruskal–Wallis test; df, degree of freedom.

**Table 6:** Analysis of descriptive statistics and analysis of age-related variations among girls.

Variable	5 (*n* = 68)		6 (*n* = 69)		7 (*n* = 84)		8 (*n* = 69)				
	Mean ± SD	KSp	Mean ± SD	KSp	Mean ± SD	KSp	Mean ± SD	KSp	χ^2^	*p*	df
Nutrition (points)	2.62 ± 0.38	0.20	2.58 ± 0.37	<0.001	2.53 ± 0.40	<0.001	2.55 ± 0.39	<0.001	2.75	0.43	3
Physical activity (points)	2.92 ± 0.57	<0.001	2.89 ± 0.46	<0.001	2.71 ± 0.54	0.09	2.69 ± 0.55	0.20	11.76	0.01	3

Abbreviations: *n*, number of subjects; M, male; F, female; SD, standard deviation; KV, coefficient of variation; KSp, level of statistical significance of Kolmogorov–Smirnov test; χ^2^, value chi squared test (Kruskal–Wallis test); *p*, level of statistical significance Kruskal–Wallis test; df, degree of freedom.

## Data Availability

Data generated and analyzed during this study are included in this article. Additional data are available from the corresponding author on request.
